# The impact of 3D-printed LAY-FOMM 40 and LAY-FOMM 60 on L929 cells and human oral fibroblasts

**DOI:** 10.1007/s00784-020-03491-2

**Published:** 2020-09-20

**Authors:** Gunpreet Oberoi, Sophie Nitsch, Klara Janjić, Hassan Shokoohi-Tabrizi, Andreas Moritz, Francesco Moscato, Ewald Unger, Hermann Agis

**Affiliations:** 1 Department of Conservative Dentistry and Periodontology, University Clinic of Dentistry, Medical University of Vienna, Sensengasse 2a, 1090 Vienna, Austria; 2 Austrian Cluster for Tissue Regeneration, Vienna, Austria; 3 Center for Medical Physics and Biomedical Engineering, Medical University of Vienna, Vienna, Austria

**Keywords:** 3D printing, Fused deposition modeling, LAY-FOMM 40 and 60, Oral fibroblasts, Microtissue spheroids, Cytotoxicity, Additive manufacturing

## Abstract

**Objectives:**

LAY-FOMM is a promising material for FDA-approved Fused Deposition Modeling (FDM) applications in drug delivery. Here we investigated the impact on oral cells.

**Materials and methods:**

We evaluated the impact of 3D-printed LAY-FOMM 40, LAY-FOMM 60, and biocompatible polylactic acid (PLA) on the activity of murine L929 cells, gingival fibroblasts (GF), and periodontal ligament fibroblasts (PDLF) using indirect (samples on cells), direct monolayer culture models (cells on samples), and direct spheroid cultures with resazurin-based toxicity assay, confirmed by MTT and Live-dead staining. The surface topography was evaluated with scanning electron microscopy.

**Results:**

The materials LAY-FOMM 40 and LAY-FOMM 60 led to a reduction in resazurin conversion in L929 cells, GF, and PDLF, higher than the impact of PLA in indirect and direct culture models. Fewer vital cells were found in the presence of LAY-FOMM 40 and 60 than PLA, in the staining in both models. In the direct model, LAY-FOMM 40 and PLA showed less impact on viability in the resazurin-based toxicity assay than in the indirect model. Spheroid microtissues showed a reduction of cell activity of GF and PDLF with LAY-FOMM 40 and 60.

**Conclusion:**

Overall, we found that LAY-FOMM 40 and LAY-FOMM 60 can reduce the activity of L292 and oral cells. Based on the results from the PLA samples, the direct model seems more reliable than the indirect model.

**Clinical relevance:**

A material modification is desired in terms of biocompatibility as it can mask the effect of drugs and interfere with the function of the 3D-printed device.

## Introduction

Fused Deposition Modeling (FDM), also termed as Fused Filament Fabrication, invented by Scott Crump in 1989, is an additive manufacturing technology applying heat for processing materials by hot-melt extrusion and injection molding [1, 2]. It is an efficient, affordable, and easy to implement 3D printing technology allowing the production of personalized devices with acceptable precision [3]. Since the expiry of the patent, the technology has been employed for affordable desktop printers, which are also used in clinics and dental offices to produce anatomical models and medical devices [4–6]. In parallel, a broad spectrum of biomimetic materials and functional structures have been developed with a variety of properties. Single materials like polymers and metals; multi-materials such as soft (TangoPlus) and hard (VeroWhite) polymers; and composites with a mineral reinforcement phase, such as hydroxyapatite, calcium carbonate, or silica, embedded in a biopolymer matrix, such as collagen or chitin, are widely available [7–9].

LAY-FOMM is a novel 3D-printed experimental material that becomes flexible and porous after immersion in water; however, the porosity is not visible to the naked eye [10, 11]. It is composed of two components: a thermoplastic elastomeric polymer component and water-soluble polyvinyl alcohol (PVA) component. After washing out PVA, the remaining material develops rubber-like and porous characteristics. There are two variants available, termed as LAY-FOMM 40 and LAY-FOMM 60, differing in their shore hardness and hence the properties, with shore hardness of A 40 and A 60, respectively. LAY-FOMM 40 has been used to adsorb and release small amounts of endogenous low-molecular-weight compounds, such as steroids, from biological matrices like plasma [12]. This is an emerging platform for the production of low-cost novel 3D-printed sorbents using an FDM printer. These materials are advantageous over solid-phase extraction and liquid-liquid extraction in terms of accuracy due to reproducibility and less carry-over, costs, and time consumption [12, 13]. Similar elastomer PVA-based 3D-printed materials like Poro-Lay find application in passive sampling devices. They offer the possibility to be designed in different thicknesses as 3D-printed membranes showing comparable results to devices integrated with standard poly (ether sulfone) membranes. They have met an expanding application research involving 3D-printed drug delivery systems with a sustained drug release in a patient-specific manner [13–16].

According to the manufacturer, the 3D LAY-FOMM 40 and 60 release no toxic compounds; however, clinical-translation requires it to be non-cytotoxic, safeguarding the macroenvironmental and cell activities. LAY-FOMM is relatively a new material, and so far, there is no scientific data supporting its biocompatibility, while a handful of studies have mentioned its role as a drug delivery agent and scaffolds in tissue regeneration. Therefore, to start with, we aimed to reveal that the response of oral cells to FDM-printed LAY-FOMM due to the potential application of these materials is dental and medical research [10, 17]. We evaluated cell viability of murine L929 fibroblasts (standard cell line for ISO 10993-5 cytotoxicity tests) [18, 19], human gingival fibroblasts (GF), and human periodontal ligament fibroblasts (PDLF) when exposed to LAY-FOMM 40 and LAY-FOMM 60 in 2D monolayer cultures and compared it with the impact of PLA. Here, the cells were either covered with the 3D-printed specimens (indirect model) or cultured on the 3D-printed material specimens (direct model) [20]. Additionally, 3D microtissue spheroid cultures of the above oral cells were used to mimic the response in a more in vivo-like setting [21–25].

## Materials and methods

### Specimen preparation

Specimens of LAY-FOMM 40 and LAY-FOMM 60 (CC-Products, Köln, Germany) were printed as discs with 12-mm diameter and 1-mm thickness on the Ultimaker 3 Extended (Ultimaker, Geldermalsen, The Netherlands). To remove polyvinyl alcohol (PVA), the samples were washed in deionized water for 1 day at room temperature. Discs with the same dimensions printed in clear polylactic acid (PLA Material Transparent, Ultimaker) served as a biocompatible comparison in the experiment.

### Preparation and cultivation of human oral fibroblasts

After tooth extraction and informed written consent given by the donors, human GF and PDLF were isolated from extracted third molars (Ethics Committee of the Medical University of Vienna, Vienna, Austria) following a previously published protocol [26, 27]. Explant cultures were done in α-minimal essential medium (α-MEM) (Invitrogen Corporation, Carlsbad, CA, USA) supplemented with 10% fetal calf serum (FCS; PAA Laboratories, Linz, Upper Austria, Austria) and antibiotics at 37 °C, 5% CO_2_, and 95% atmospheric moisture. For the experiments both GF and PDLF were seeded at 50,000 cells/cm^2^ and incubated for 24 h.

### Cultivation of L929 cells

L929 cells from adipose tissue fibroblasts were used as it is a standard cell line for ISO 10993-5 cytotoxicity tests. L929 cells were cultured in α-MEM (Invitrogen Corporation) supplemented with 10% FCS (PAA Laboratories) and antibiotics at 37 °C, 5% CO_2_, and 95% atmospheric moisture. For the experiments, L929 cells were seeded at 50,000 cells/cm^2^ and incubated for 24 h.

### Indirect culture model

GF, PDLF, and L929 cells were seeded in 24 well culture plates and covered with disc-shaped specimens of LAY-FOMM 40, LAY-FOMM 60, and PLA. Then, cells were subjected to resazurin-based toxicity assays. Furthermore, Live-dead staining was performed. Unexposed 2D cell cultures and those treated with staurosporine served as a positive and negative control, respectively.

### Direct culture model

GF, PDLF, and L929 cells were seeded onto disc-shaped specimens of LAY-FOMM 40, LAY-FOMM 60, and PLA for 24 h. Then cells were subjected to MTT and resazurin-based toxicity assays. Live-dead staining was performed to confirm the results from the above-mentioned cytotoxicity assays. Unexposed 2D cell cultures and those treated with staurosporine served as a positive and negative control, respectively.

### Spheroid direct culture model

To build three-dimensional microtissue spheroids of GF and PDLF, we procured flexible 3D Petri Dish® (Microtissues Inc., Providence, RI, USA) with 35 spheroidal recesses and poured 330 μl molten agarose (2% agarose powder in 0.9% NaCl) into the molds, under aseptic conditions. After 2 min of gelling, the molds were inverted into alpha-MEM growth medium for conditioning up to 5 min. Molds were carefully transferred into the 24 well plates (TPP Techno Plastic Products, Trasadingen, Switzerland) using sterile forceps, and each mold received 75 μl of cell suspension of GF and PDLF with a cell count of 73 × 10^5^ cells/ml. L929 cells do not form spheroids; hence, they were excluded from this step. Cell settling time of 15 min was followed by the addition of fresh α-MEM 1 ml/well, outside the mold. The plate was incubated for 24 h at 37 °C, and microscopic evaluation confirmed the formation of spheroids over 24 h. Spheroids were cultured directly on LAY-FOMM 40 and LAY-FOMM 60 for the next 24 h. The set-up was then subjected to resazurin-based toxicity assay and MTT and Live-dead staining.

### Resazurin-based cytotoxicity assay

A resazurin-based cytotoxicity assay was done according to the instructions of the manufacturer. Resazurin dye solution (Sigma-Aldrich, Germany) was added in an amount equal to 10% of the culture medium and incubated at 37 °C for 4 h in monolayer culture models (direct and indirect models) and for 8 h in a spheroid culture model of GF and PDLF. Fluorescence was evaluated using a Synergy HTX Multi-Mode Reader (BioTek, Winooski, VT, USA) at a wavelength of 600 nm, using an excitation wavelength of 540 nm. Untreated 2D and 3D cell cultures and those treated with staurosporine served as a positive and negative control, respectively. Four independent experiments were performed for 2D and spheroid cell cultures.

### MTT staining

Indirect and direct cultures were incubated with 1 mg/mL MTT (3-(4,5-Dimethylthiazol-2-yl)-2,5-Diphenyltetrazolium Bromide, Sigma-Aldrich, St. Louis, MO, USA) at 37 °C for 2 h. Formazan crystal formation was observed under a light microscope, and images were taken at 20-fold magnification. Unexposed 2D cell cultures and those treated with staurosporine served as a positive and negative control, respectively.

### Live-dead staining

GF and PDLF cultures were stained with Live-Dead Cell Staining Kit (Enzo Life Sciences AG, Lausen, TX, USA) according to the instructions by the manufacturer. Cultures were evaluated using fluorescence microscopy for green and red dyes, with a B-2A filter (excitation filter wavelengths, 450–490 nm). Vital cells appeared green, while dead cells appeared red in indirect cell cultures. Images were taken at 100-fold magnification. Unexposed 2D cell cultures and those treated with staurosporine served as a positive and negative control, respectively.

### Scanning electron microscopy

Scanning electron microscopy (SEM) images of the 3D-printed materials LAY-FOMM 40 and 60 and PLA were generated utilizing the Quanta 200 system (FEI Company, USA). The samples were mounted on an aluminum sample holder and sputtered on both sides for the same time with a 10-nm-thick gold layer using the EM ACE200 sputtering device (Leica, Germany). Then images were taken at an accelerating voltage of 15 kV in SE mode at 2000-fold magnification.

### Statistical analysis

Statistical analysis was performed with IBM SPSS Statistics Version 23 (IBM Corporation, Armonk, NY, USA) using the Kruskal-Wallis-test post hoc Mann-Whitney-test. The level of significance was set at *p* < 0.05.

## Results

### Activity of L929 and oral fibroblasts in response to LAY-FOMM 40 and LAY-FOMM 60 in an indirect cell culture model

**Fig. 1:**
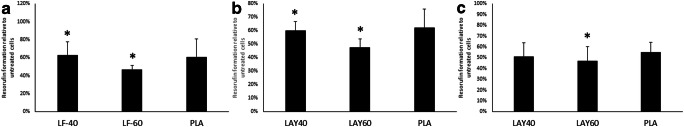
Response of L929, gingival fibroblasts, and periodontal ligament fibroblasts activity to printed LAY-FOMM 40, LAY-FOMM 60, and PLA in an indirect cell culture model. L929 cells (**a**), gingival fibroblasts (GF, **b**), and periodontal ligament fibroblasts (PDLF, **c**) were exposed to LAY-FOMM 40 (LF-40) and LAY-FOMM 60 (LF-60) specimens. Then, cells were subjected to resazurin-based toxicity assays Data are given as mean + standard deviation and are presented relative to the untreated control. At least four independent experiments were performed. * *p* < 0.05 vs. untreated control

**Fig. 2:**
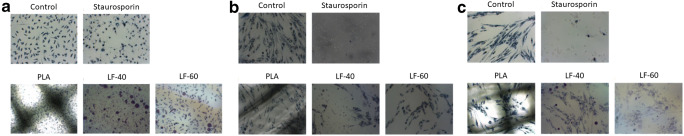
MTT staining of L929, gingival fibroblasts, and periodontal ligament fibroblasts activity to printed LAY-FOMM 40, LAY-FOMM 60, PLA, control, and staurosporine in an indirect cell culture model. L929 cells (**a**), gingival fibroblasts (GF, **b**), and periodontal ligament fibroblasts (PDLF, **c**) were exposed to LAY-FOMM 40 (LF-40) and LAY-FOMM 60 (LF-60) specimens. MTT staining was performed

**Fig. 3:**
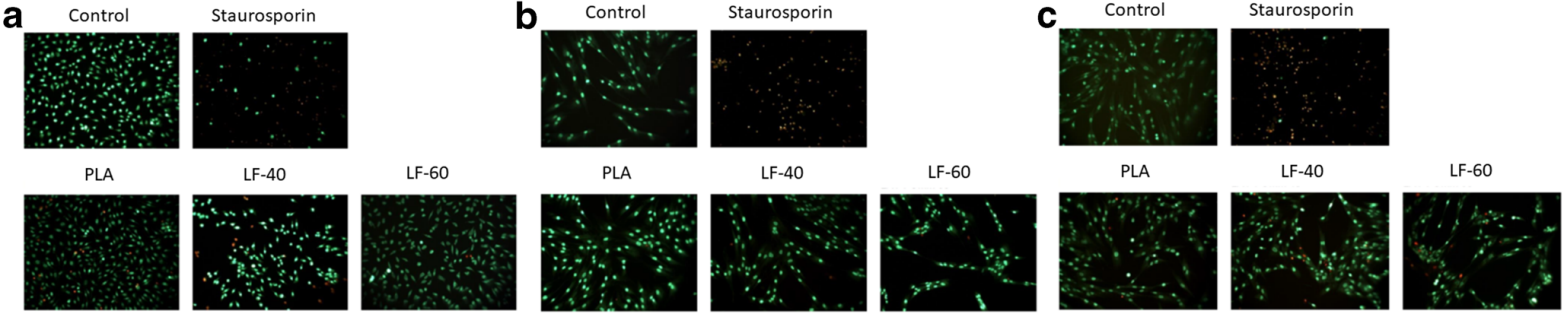
Live-dead staining of L929, gingival fibroblasts, and periodontal ligament fibroblasts in response to LAY-FOMM 40, LAY-FOMM 60, PLA, control, and staurosporine in an indirect monolayer cell culture model. L929 cells (**a**), gingival fibroblasts (GF, **b**), and periodontal ligament fibroblasts (PDLF, **c**) were exposed to LAY-FOMM 40 (LF-40) and LAY-FOMM 60 (LF-60) specimens. Live-dead staining was performed

L929 cells, GF, and PDLF were exposed to LAY-FOMM 40, LAY-FOMM 60, and PLA specimens. Cells were subjected to resazurin-based toxicity assays. Furthermore, MTT staining and Live-dead staining were performed. When exposed to LAY-FOMM 40, L929, GF, and PLDF showed 63%, 60%, and 51% resorufin formation compared with untreated cells, respectively. Exposure of L929, GF, and PLDF to LAY-FOMM 60 led to resorufin formation 47%, 48%, and 47% relative to untreated cells, respectively. Treatment with PLA leads to 61%, 62%, and 55%, respectively (Fig. 1). Overall, LAY-FOMM 60 showed the lowest levels of resorufin formation with all cells. Vital blue and green cells were visible in the MTT staining (Fig. 2) and the Live-dead staining (Fig. 3) for LAY-FOMM 40 and 60 as well as PLA for all tested cell types.

### Activity of L929 and oral fibroblasts in response to LAY-FOMM 40 and LAY-FOMM 60 in a direct monolayer culture model

**Fig. 4:**
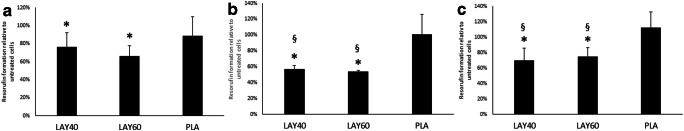
Response of L929, gingival fibroblasts, and periodontal ligament fibroblasts activity to printed LAY-FOMM 40, LAY-FOMM 60, and PLA in a direct cell culture model. L929 cells (**a**), gingival fibroblasts (GF, **b**), and periodontal ligament fibroblasts (PDLF, **c**) were cultured on LAY-FOMM 40 (LF-40) and LAY-FOMM 60 (LF-60) specimens directly. Then, cells were subjected to resazurin-based toxicity assays. Data are given as mean + standard deviation and are presented relative to the untreated control. At least four independent experiments were performed. *p < 0.05 vs. untreated control. §*p* < 0.05 vs. PLA

**Fig. 5:**
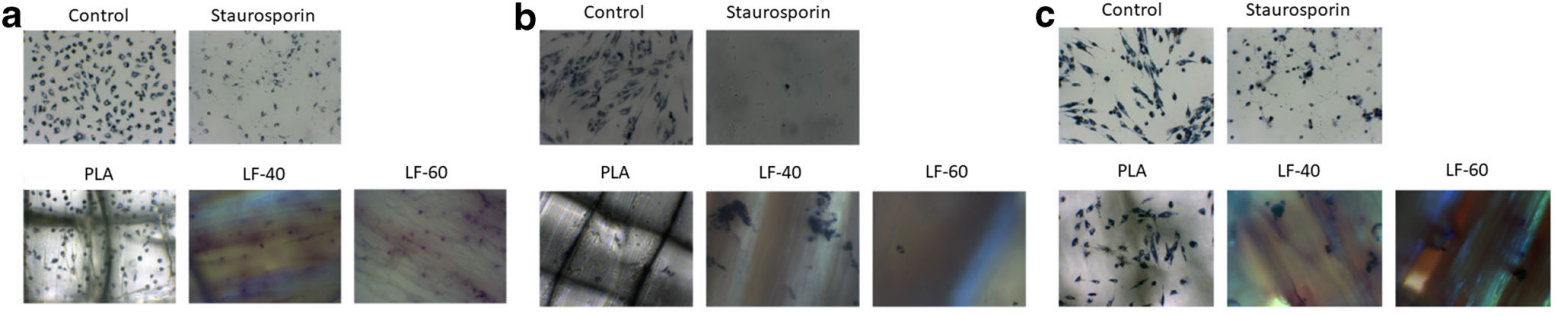
MTT staining of L929, gingival fibroblasts, and periodontal ligament fibroblasts activity to printed LAY-FOMM 40, LAY-FOMM 60, PLA, control, and staurosporine in a direct cell culture model. L929 cells (**a**), gingival fibroblasts (GF, **b**), and periodontal ligament fibroblasts (PDLF, **c**) were cultured on LAY-FOMM 40 (LF-40) and LAY-FOMM 60 (LF-60) specimens. MTT staining was performed

**Fig. 6:**
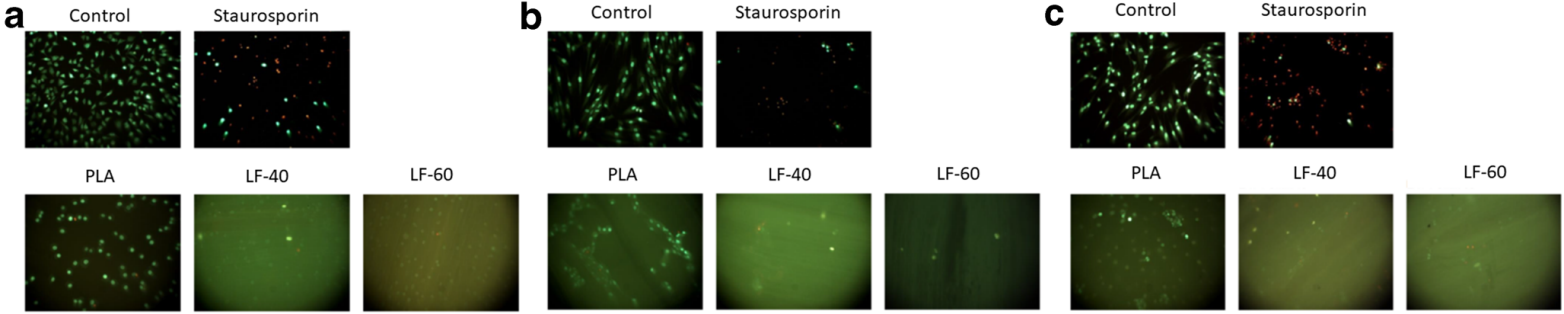
Live-dead staining of L929, gingival fibroblasts, and periodontal ligament fibroblasts in response to LAY-FOMM 40, LAY-FOMM 60, PLA, control, and staurosporine in a direct monolayer cell culture mode. L929 cells (**a**), gingival fibroblasts (GF, **b**), and periodontal ligament fibroblasts (PDLF, **c**) were cultured directly on LAY-FOMM 40 (LF-40) and LAY-FOMM 60 (LF-60) specimens. Then, Live-dead staining was performed

L929 cells and oral fibroblasts were exposed to PLA, LAY-FOMM 40, and LAY-FOMM 60 specimens. Cytotoxicity was evaluated with resazurin-based toxicity assays. In addition, MTT staining and Live-dead staining were done. When treated with LAY-FOMM 40, L929, GF, and PLDF showed 76%, 57%, and 70% resorufin formation compared with untreated cells, respectively. Exposure of L929, GF, and PLDF to LAY-FOMM 60 led to resorufin formation 66%, 54%, and 75% relative to untreated cells, respectively. Treatment with PLA leads to 89%, 101%, and 112%, respectively (Fig. 4). Also, in the direct model, LAY-FOMM 60 showed the lowest levels of resorufin formation with all cells. Overall, the levels of resorufin formation were higher for LAY-FOMM 40 and PLA in the direct model. Vital blue and green cells were visible in the MTT staining (Fig. 5) and the Live-dead staining (Fig. 6) for LAY-FOMM 40 and 60 as well as PLA for all tested cell types.

### Response of oral fibroblasts to LAY-FOMM 40 and LAY-FOMM 60 in a direct spheroid culture model

**Fig. 7:**
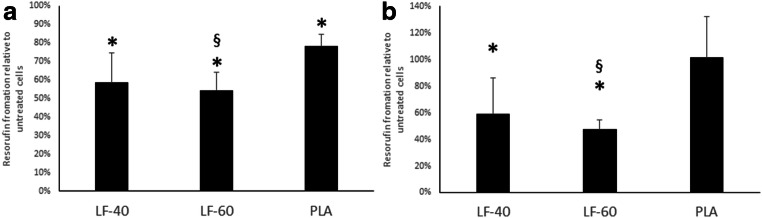
The response of gingival fibroblasts and periodontal ligament fibroblasts to LAY-FOMM 40, LAY-FOMM 60, and PLA in a direct spheroid culture model. Spheroids of gingival fibroblasts (GF, **a**) and periodontal ligament fibroblasts (PDLF, **b**) were seeded and cultured on LAY-FOMM 40 (LF-40) and LAY-FOMM 60 (LF-60) and PLA. Then, cells were exposed to resazurin-based toxicity assays. Data are given as mean ± standard deviation and are presented relative to the untreated control. Four independent experiments were performed. *p < 0.05 vs. untreated control. §*p* < 0.05 vs. PLA

Spheroids of GF and PDLF were cultured directly on the 3D-printed discs of LAY-FOMM 40, LAY-FOMM 60, and PLA. The cultures underwent resazurin cytotoxicity assay and the material comparison uniformly showed that the LAY FOMM 60 was more cytotoxic than LAY FOMM 40 relative to the positive control in both GF and PDLF spheroids. PLA slightly decreased the vitality of GF spheroids but had no cytotoxic impact on PDLF spheroids. GF and PDLF exposed to LAY-FOMM 40 showed 59% and 59% of resorufin formation relative to control, respectively, while GF and PDLF exposed to LAY-FOMM 60 reached 54% and 47% of control, respectively. Treatment of GF and PDLF with PLA resulted in 78% and 101% resorufin formation relative to untreated cells (Fig. 7).

### Scanning electron microscopic evaluation of the printed LAY-FOMM 40, LAY-FOMM 60, and PLA specimens

**Fig. 8:**
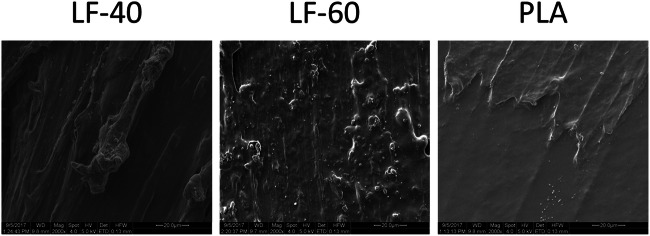
Scanning electron microscopic evaluation of the printed LAY-FOMM 40, LAY-FOMM 60, and PLA specimens

Scanning electron microscopy displayed that surface topography of printed LAY- FOMM 60 specimens were more granular, irregular, and porous than LAY-FOMM 40, while PLA appeared planar compared with all, considering the fact that all specimens were printed using the same printer settings (Fig. 8).

## Discussion

3D printing is anticipated to be one of the most advanced remedies for personalized medicine and dentistry. This technology has the potential to develop biomedical devices with intricate geometries and surface characteristics. In personalized medicine, the patient receives a tailored dose and release profile based on his pharmacokinetics data and FDM is an appropriate aid for this emerging field [28]. An interdisciplinary methodology coalescing cell biology, material science, biomedical engineering, and pre-clinical evaluation is essential for addressing the growing technology of additive manufacturing for successful clinical translation. In the development of such precision biomaterials and biomedical devices, 3D printing promises to deliver highly reproducible and constant pore size and geometry, which can be tailored to replicate the target tissue characteristics [29]. We have known that resin-based dental materials are not completely inert on exposure to the oral cells and may release non-polymerized components in the long term, which could be degradable or non-degradable [30, 31]. Since 3D-printed materials are now replacing various conventional technologies with the possibility of being implanted into the human body, concerns regarding their potential cytotoxicity are justified.

With the increasing application of low-cost FDM printers in day-to-day dentistry, and the introduction of a versatile material like LAY-FOMM, it was interesting to investigate its cytotoxic effect on oral cells before clinical translation. The in vitro results in our study presented that PDLF and GF are responsive to LAY-FOMM 40 and 60 by undergoing a reduction in resazurin conversion, which was higher than the impact of PLA in the indirect and direct culture model. Fewer vital cells were found in the presence of LAY-FOMM 40 and LAY-FOMM 60 than PLA in MTT staining and Live-dead staining in both indirect and direct culture models. Similar to monolayer cultures, spheroid microtissue cultures also showed a greater reduction of cell activity of GF and PDLF with LAY-FOMM 40 and 60 as compared with PLA. Here, we report the first use of two “oral” cell lines in a 3D cell culture model in the presence of LAY-FOMM 40 and 60 that has already been used as a prospective vehicle for drug delivery, sorbent, and a sampling device [12, 13, 16]. These results are supported by our findings on the response of L929 cells, which are the cell line recommended for cytotoxicity tests in ISO 10993-5. Hence the outcome of this study is highly relevant clinically. The reason for choosing PLA as a comparison is because it is often used in biocompatible implantable constructs, including other materials like polyglycolic acid (PGLA), polycaprolactone (PCL), and combinations of polyethylene glycol (PEG) [24]. PLA and PLA-polymer fusions in amalgamation with various hydrogels have been shown to be efficient cartilage regeneration scaffolds [29, 32, 33]. Hollow bullet-shaped implants with PLA coating have been developed as the drug release from the implant can be regulated by changing pore size, type of matrix, and coating thickness [34].

The decrease in the resazurin conversion by GF and PDLF as well as L929 in the presence of LAY-FOMM 40 and 60 can be attributed to the released components from incomplete polymerization of the 3D-printed materials [35].

Interestingly, the three applied culture models lead to different results. While using indirect samples on cells can show higher toxicity of the samples, the direct monolayer culture models (cells on samples) did not show such a pronounced impact. This might be due to the physical irritation of the cells due to the presence of the material in the indirect model. The cells in 3D direct spheroid cultures were less susceptible to potential negative effects of the LAY-FOMM 40 and 60. This highlights the importance of the relevant culture mode. This is in line with previous studies evaluating the cytotoxicity of 3D printing material [31].

Many researchers have credited the effects of extrusion process factors such as the speed of 3D dispensing and filament feed, melt viscosity, pressure and temperature gradients, nozzle design, shear-thinning, crystallization rate, the addition of stabilizers and other additives, path-planning, and part orientation for the biomechanical and physical properties of the 3D-printed materials [36]. Layer resolution of the Ultimaker 3 Extended (Ultimaker, Geldermalsen, The Netherlands) printer used in our study is 60–150 μm for a 0.25-mm nozzle, 20–200 μm for a 0.4-mm nozzle, and 20–600 μm for a 0.8-mm nozzle. The build speed of the printer is < 24 mm^3^/s, and print head travel speed is 30 to 300 mm/s. It has been shown that the specimen porosity, surface area, mechanical properties, diffusion, and fluid flow rates may affect cell seeding, adherence, and growth. Additionally, the layer resolution of the printing process may govern the above-mentioned parameters owing to the limited nozzle size between 0.25 and 0.8 mm [37, 38]. It is mandatory to regulate these factors for a desirable and controlled release of chemicals from the 3D-printed specimens [37, 38]. In the present paper, we applied a 0.4-mm nozzle.

Our findings are however slightly contradictory to the research by Ahanger et al., where it was shown that unloaded LAY-FOMM 40 and 60 scaffolds had no effect on the metabolic activity and proliferation of prostate cancer cell line LAPC4 and patient-derived spine metastasis cells as against the doxorubicin drug-loaded scaffolds [16]. Another reason for the lower level of resorufin conversion could be the presence of residual polyvinyl alcohol (PVA), which is a water-soluble preliminary constituent of LAY-FOMM 40 and 60. Its properties like hot-processability, availability in different molecular weights, and water-induced shape memory behavior make it appropriate for 3D printing [39]. But it has been shown to have a low toxic response in implanted rats [40]. However, dedicated biocompatibility tests for LAY-FOMM have not been performed so far. Hence, it appears that the material might require chemical modification to make it more biocompatible. In parallel to the progress in new 3D-printed materials for applications like drug delivery, sampling devices, and sorbents, material fabrication protocols need to be transformed; otherwise, it might affect the release and absorption kinetics. Such advancements will make the functionality of the 3D-printed device more precision-oriented. 3D printing provides an option of manipulating the spatial distribution within a defined polymer composition instead of developing a new material to bring a change in the above-mentioned parameters and characteristics. It has unprecedented reproducibility, high throughput, versatility, and accuracy [41, 42].

The ease of modification of the structural characteristics like pore size in FDM printing technology is very important for clinical translation of the functionality of the materials. For example, for bone tissue engineering, the critical pore size of scaffolds is about 100 μm [28, 29], while optimum pore size for bone growth is about 300–350 μm [30], and > 500 μm promotes soft tissue ingrowth [31, 32]. In this way, FDM technology can be used to produce materials with macro-scale to the nano-scale internal geometrical characteristics [43]. The first 3D-printed tablet (Spritam®) has met the FDA requirements, claiming the commercial vitality of FMD technique [41]. Another field where LAY-FOMM can find application is the manufacture of 3D-printed masks with pore size ranging from 16.90 to 146.60 μm [44] as additive manufacturing gives the opportunity to manipulate the pore size by changing the printer settings [45]. Our results do not oppose the use of LAY-FOMM 40 and 60 in medical and dental applications, but it is suggested to modify the material processing protocol to not interfere with cell metabolism and viability.

Potential applications of these experimental materials could be in the form of nicotine patches for controlled release of nicotine into the system. Additionally, they can be used as local chemotherapeutic agents for oral and other tumors based on the 3D imaging data of the tumor. This can help reduce the size of the tumor prior to resection and prevent strong side effects associated with systemic drug delivery. Another prospective application could be in the form of root canal-specific 3D-printed medicaments to avoid unnecessary apical extrusion of the material, which is often toxic to the periapical cells. Additionally, with the possibility of designing the porosity, pore size, and porous structure, it can be used to manufacture face masks against specific aerosols, bacteria, and viruses. Hence, porous 3D-printed scaffolds may provide a novel and affordable methodology to locally deliver chemotherapeutics or function as sorbent devices in a customized manner with a modified composition [16].

## Conclusion

LAY-FOMM 40 and LAY-FOMM 60 can reduce the activity of L292 and oral cells. Based on the results from the PLA samples, the direct model seems more reliable than the indirect model. With a great potential in pharmaceutical development, FDM in general is still confronted by challenges like material characteristics and cytotoxicity. Further advancement of equipment, by regulating the manufacturing parameters and optimization of polymeric formulations, is imperative for a successful clinical translation.

## Abbreviations

CAD: Computer-aided design
CAM: Computer-aided manufacturing
FCS: Fetal calf serum
FFF: Fused Filament Fabrication
GF: Gingival fibroblasts
MTT: (3-(4,5-Dimethylthiazol-2-yl)-2,5-Diphenyltetrazolium Bromide
SEM: Scanning electron microscopy
SLA: Stereolithography
SLS: Selective laser sintering
α-MEM: α-Minimal essential medium
